# Highly Linear Perovskite Synapses for Neuromorphic Computing

**DOI:** 10.1002/advs.78461

**Published:** 2026-10-26

**Authors:** Zhongwen Sun, Boyu Wan, Zifan Zhang, Zhikai Wang, Xuan Zhao, Haonan Si, Qingliang Liao, Yue Zhang

**Affiliations:** ^1^ Academy for Advanced Interdisciplinary Science and Technology Key Laboratory of Advanced Materials and Devices for Post‐Moore Chips Ministry of Education University of Science and Technology Beijing Beijing P. R. China; ^2^ Beijing Key Laboratory of Two‐Dimensional Semiconductors for Future Chips School of Materials Science and Engineering State Key Laboratory for Advanced Metals and Materials University of Science and Technology Beijing Beijing P. R. China

**Keywords:** artificial synapses, halide perovskites, memristors, neuromorphic computing

## Abstract

Neuromorphic computing offers a compelling route to data‐intensive applications with boosted computing power and energy efficiency. However, its accuracy for edge vision tasks is critically bottlenecked by the inherent non‐linearity and asymmetry of conductance updates in analog memristive synapses. Here, we report a highly linear memristive synapse based on vertically aligned alternating cations in the interlayer space phase halide perovskite. A synergistic regulation strategy involving additive engineering and solvent atmosphere annealing is introduced to fabricate GAMA_5_Pb_5_I_16_ halide perovskite films with vertical crystal orientation. The resulting films facilitate controllable ion migration, allowing the fabricated devices to achieve an ultra‐low energy consumption of 5.5 aJ alongside 100 discrete conductance states with non‐linearity as low as 0.027 and 0.064 and asymmetry of 0.037. These attributes enable the synapses to perform object detection tasks with an mAP50 of 57.6%, retaining 98.1% of the floating‐point baseline performance. This work provides a promising biomimetic solution for efficient visual perception in intelligent transportation, paving the way for high‐precision neuromorphic systems.

## Graphical abstract

Voltage spikes applied to an Au/ACI‐phase perovskite/ITO memristor emulate the ionic signaling of a biological synapse, generating a tunable postsynaptic current. The vertically aligned perovskite film confines iodide‐vacancy migration, enabling highly linear, and symmetric conductance updates with 100 states and 5.5 aJ per event. Such synapses sustain accurate object detection, offering an energy‐efficient route to neuromorphic vision.

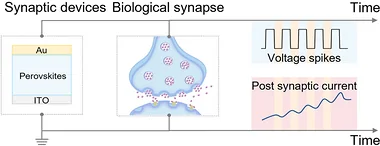

## 1 Introduction

Rapidly evolving artificial intelligence has generated an urgent demand for computing systems to handle data‐intensive tasks [1]. While traditional von Neumann architectures suffer from the physical segregation of memory and processing units, leading to frequent data transfers. This structural constraint results in significant latency, high power consumption, and substantial computational overhead [2, 3]. To address these limitations, near‐sensor neuromorphic computing architectures have emerged as a promising paradigm [4, 5]. By integrating memory and processing functions within a single unit, this architecture facilitates in situ data processing near the sensors [6, 7].

Artificial synaptic devices, characterized by integrated memory and computing capabilities, serve as the essential components for neuromorphic computing architectures [8]. Operating as high‐performance hardware accelerators, these devices directly process analog sensor outputs without the need for analog‐to‐digital conversion [9]. Leveraging Ohm's Law and Kirchhoff's Law, synaptic devices can perform vector‐matrix multiplication between input voltage vectors and conductance matrices in a single step. This inherent parallelism minimizes redundant data transmission between components, fundamentally enhancing computational efficiency [10]. Artificial synaptic devices primarily include two‐terminal memristors and three‐terminal transistors. Among them, two‐terminal (2T) memristor‐type artificial synapses utilize electrical pulses as programming stimuli to achieve high‐density physical integration, naturally aligning with the operational mechanisms of biological synapses [11].

Despite their potential, achieving multi‐level tunable and highly linear conductance states remains a significant challenge for memristor‐type artificial synapses. Unexpected conductance updates can degrade the performance of vector‐matrix multiplication, subsequently affecting the training and recognition accuracy of artificial neural networks [12]. Addressing these non‐idealities requires leveraging the intrinsic physical phenomena of materials for precise modulation [13]. In this context, halide perovskites provide an ideal platform. As mixed ionic‐electronic conductors, halide perovskites exhibit diverse dynamic responses to external stimuli, enabling the precise emulation of biological synaptic plasticity through multi‐state conductance tuning [14, 15]. These materials possess high defect tolerance and low point‐defect formation energies, maintaining a stable crystal structure even at high defect densities [16, 17]. Furthermore, the low activation energy for ion migration enables memristive effects driven by conductive filament formation or interfacial barrier modulation [18]. Moreover, ion concentration and mobility can be regulated via compositional engineering and external stimuli, making halide perovskites excellent candidates for artificial synapses [19]. However, the polycrystalline nature and high defect densities of these materials often lead to a lack of effective control over ion migration during voltage programming [20]. The inherently stochastic nature of ion migration typically induces abrupt conductance changes, impeding the linear and symmetric weight update process in neural networks, and thereby limiting the accuracy of neuromorphic tasks [12, 21].

To address these challenges, we rationally design a highly linear two‐terminal memristive synapse based on vertically aligned alternating cations in the interlayer space (ACI) phase of a two‐dimensional (2D) halide perovskite. By synergistically combining additive engineering with solvent‐atmosphere annealing, we achieve oriented crystallization of GAMA_5_Pb_5_I_16_ films, which enables precise control over vertical ion transport pathways. The resulting synaptic devices exhibit exceptional linearity and symmetry in multi‐level conductance modulation, alongside ultralow switching energy per synaptic event. In addition, an 8 × 8 crossbar halide perovskite synaptic array is constructed with high device yield. System‐level simulations based on the proposed synapses demonstrate the successful execution of complex vision tasks, including object detection, segmentation, and perception‐driven classification. This work highlights the potential of halide perovskite synapses in neuromorphic computing, while laying the groundwork for their broader application in autonomous driving, environmental monitoring, and industrial safety.

## 2 Results and Discussion

### 2.1 Vertically Aligned Halide Perovskite Film

To achieve highly linear and symmetric synaptic devices, the halide perovskite is targeted to possess a vertical orientation relative to the bottom electrode within a smooth, pinhole‐free film because grains with non‐vertical orientations can impede ion migration [20, 22]. In order to enhance their crystallinity, a synergistic regulation strategy involving additive engineering, and isopropanol (IPA) solvent atmosphere annealing (referred to as MACl‐IPA treatment) was proposed to fabricate ACI‐phase GAMA_5_Pb_5_I_16_ films. For the pristine samples, the precursor solution was spin‐coated directly onto the substrate followed by annealing. In contrast, for the vertically oriented films, MACl was incorporated into the precursor solution as an additive, and the substrate was exposed to an IPA atmosphere during the annealing process (further details are provided in the Experimental Section). MACl‐IPA treatment can regulate the crystallization rate of the film, thereby promoting the vertical orientation of the film.

The morphologies of the resulting films were characterized using scanning electron microscopy (SEM), as shown in Figure 1a–c. Compared to the pristine sample, the original ACI‐phase 2D perovskite film exhibits smaller grain sizes and a higher density of grain boundaries. As discontinuous interfaces, these grain boundaries serve as vertical diffusion pathways that facilitate the infiltration of moisture and oxygen, potentially accelerating the degradation of the perovskite from the surface into the bulk [23]. In contrast, the morphology of the film significantly improved following MACl‐IPA treatment. The optimized perovskite film displays substantially larger grain sizes with continuous boundaries and virtually no detectable surface pores. The cross‐sectional SEM images of the pristine and treated films are shown in the insets of Figure 1b,c, respectively. The pristine film shows a non‐uniform cross‐section with voids and pinholes distributed at the interfaces and grain boundaries. Conversely, the regulated perovskite film possesses dense, large grains that facilitate efficient charge transport [24]. Furthermore, atomic force microscopy (AFM) images (Figure S1) confirm a significant increase in grain size after MACl‐IPA treatment. To further verify the compositional uniformity of the film after the additive treatment, cross‐sectional energy‐dispersive X‐ray spectroscopy (EDS) elemental mapping was performed, as shown in Figure S2. The results demonstrate that Pb and I elements are uniformly distributed across the entire thickness of the film.

**FIGURE 1:**
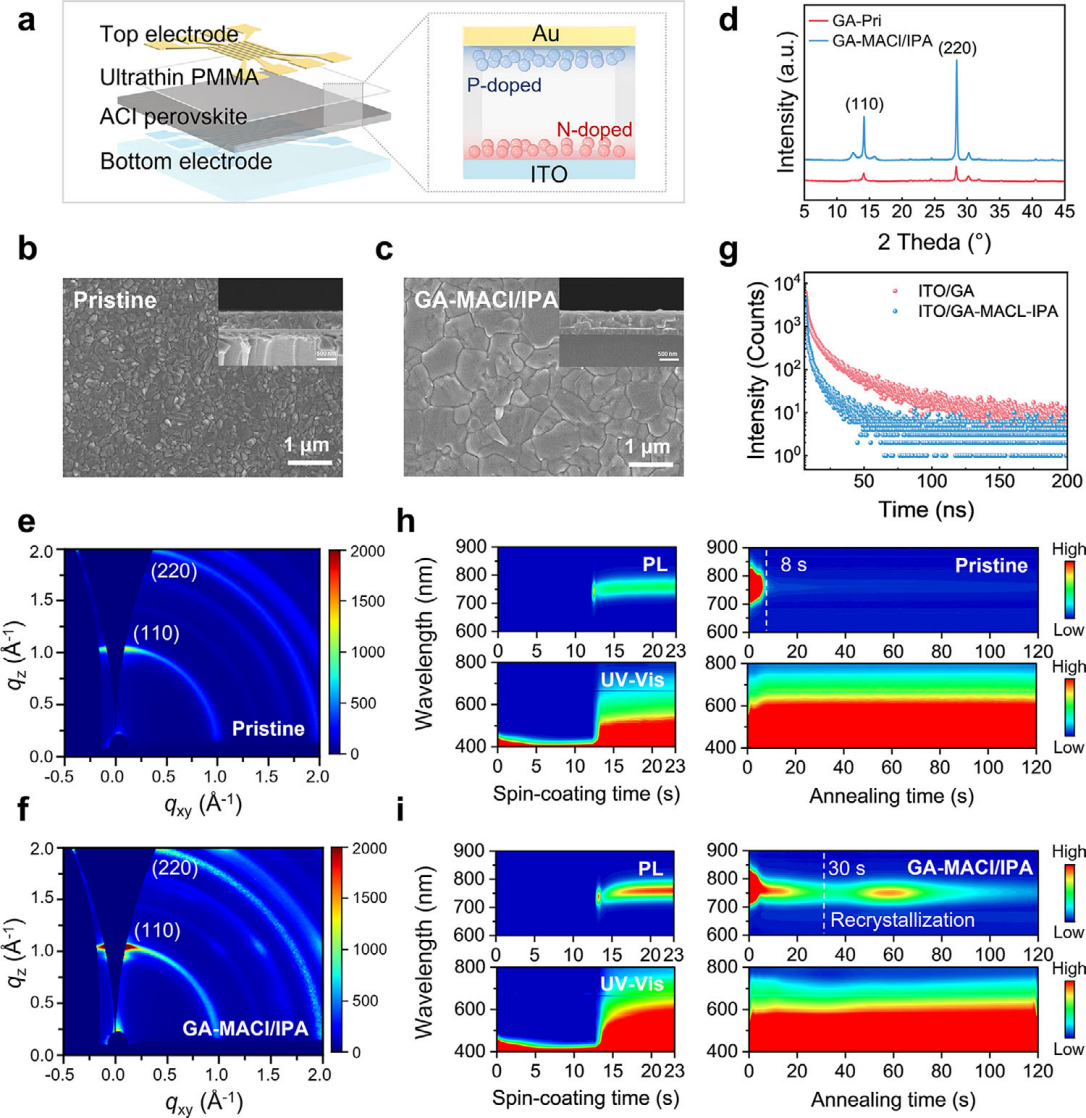
Material properties of GAMA_5_Pb_5_I_16_ perovskite films. (a) Schematic illustration of the 2D halide perovskite memristor device architecture. (b, c) Top‐view and cross‐sectional (insets) scanning electron microscopy (SEM) images of the pristine and MACl‐IPA‐treated films. (d) X‐ray diffraction (XRD) spectra of the pristine and MACl‐IPA‐treated films. (e, f) Grazing‐incidence wide‐angle X‐ray scattering (GIWAXS) spectra of the pristine and MACl‐IPA‐treated films. (g) Time‐resolved photoluminescence (TRPL) spectra of the pristine and MACl‐IPA‐treated films. (h, i) In situ PL (top) and in situ UV–vis absorption (bottom) spectra over time for the pristine and MACl‐IPA‐treated films during the spin‐coating (left panels) and thermal annealing (right panels) processes.

To investigate the crystal orientation of 2D halide perovskite films, X‐ray diffraction (XRD) analysis was further employed before and after MACl‐IPA treatment. Figure 1d presents the XRD spectra of the ACI‐phase 2D perovskites. The MACl‐IPA treated 2D perovskite film exhibits intense diffraction peaks at 14.21° and 28.71°, corresponding to the (110) and (220) lattice planes, respectively [25]. These prominent peaks demonstrate the vertical alignment of the inorganic and organic layers relative to the substrate. In contrast, the pristine film shows significantly lower intensities at these specific angles of 14.21° and 28.71°, while exhibiting relatively stronger peaks near 24.5° and 31.9°, which are indexed to the (202) and (310) lattice planes of the 3D perovskite phase [26]. These results indicate that the untreated film possesses a random orientation and exhibits polycrystalline characteristics with low crystallinity. In contrast, 2D perovskite films after MACl‐IPA treatment demonstrated vertical orientation and enhanced crystallinity. To further elucidate the crystal orientation on the substrate, grazing‐incidence wide‐angle X‐ray scattering (GIWAXS) measurements were performed, as shown in Figure 1e,f. The untreated film displays distinct diffraction rings corresponding to the (110) and (220) lattice planes, indicating a high degree of random polycrystalline orientation, which aligns with the XRD findings. However, the MACl‐IPA‐treated film exhibits intense, discrete Bragg spots, signifying superior crystallinity and a strong preferred orientation relative to the substrate. Notably, these diffraction signals are concentrated along the qz axis, confirming the vertical crystal orientation of the perovskite film [27, 28]. These results indicate that the MACl‐IPA treatment transforms the film into a textured film composed of highly crystalline, vertically oriented nanocrystals, whereas the pristine film consists of poorly crystalline grains with nearly random orientations.

To investigate carrier transfer dynamics, steady‐state photoluminescence (PL) spectra were measured for the 2D perovskite films, both before and after MACl‐IPA treatment, deposited on ITO substrates (Figure S3). The MACl‐IPA treated film exhibits significantly lower PL intensity compared to the pristine film. This reduction indicates that charge carriers in the 2D perovskite can be more efficiently transferred to the substrate along the vertically aligned inorganic layers. Furthermore, time‐resolved photoluminescence (TRPL) spectra were also analyzed. As shown in Figure 1g, the treated film displays a faster decay time than the pristine film. The calculated average carrier lifetime decreased from 2.87 to 1.29 ns, representing a substantial reduction of approximately 55%. This result further confirms that the vertically oriented conductive inorganic framework facilitates carrier extraction from the perovskite film to the ITO substrate [13]. Such vertical alignment establishes high‐efficiency carrier transport pathways between the top and bottom electrodes, which is highly advantageous for synaptic device performance. Ultraviolet‐visible (UV–vis) absorption spectra of the treated films were obtained, as shown in Figure S4. It can be observed that halide perovskite films clearly exhibit the absorption edge of the 3D phase, alongside distinct exciton absorption bands at approximately 621, 652, and 678 nm. The Tauc plot indicated the optical bandgap (Eg) was calculated to be 1.58 eV, confirming that the MACl‐IPA treatment preserves the integrity of the basic bandgap structure [29].

To explore the mechanism underlying the MACl‐IPA treatment in regulating GAMA_5_Pb_5_I_16_ crystallization, X‐ray photoelectron spectroscopy (XPS) was performed (Figure S5). Notably, no Cl 2p orbital peaks were detected near 198.7 eV in either film, indicating that Cl is not incorporated into the final perovskite lattice. However, the Pb 4f and I 3d orbital peaks in the XPS spectra of the treated film show distinct shifts compared to the control film. After MACl‐IPA treatment, the Pb 4f_5/2_ and Pb 4f_7/2_ peaks shifted from 143.2 and 138.4 eV to 142.9 and 138.0 eV, respectively. This shift toward lower binding energy suggests a reduction in uncoordinated Pb^2+^ within the film, likely due to the passivation of n‐type defects by MACl [30]. The electron density of Pb increases as a result of the underlying electron donation from MACl [31]. Additionally, the I 3d_3/2_ and 3d_5/2_ peaks shifted from 630.5 and 618.9 eV to 630.6 and 619.1 eV, respectively. This positive shift of the I_3d_ peaks may be attributed to the passivation of Pb‐I antisite defects by MACl [32].

To reveal the underlying film formation mechanism and understand the crystallization kinetics, in situ PL and in situ UV–vis absorption spectroscopy were performed (Figure 1h,i). The perovskite crystallization process was monitored throughout the spin‐coating and thermal annealing stages. Regarding the spin‐coating process, a strong PL peak emerges near 750 nm when the antisolvent is dripped at approximately 12 s, signifying the rapid nucleation, and formation of the halide perovskite [33]. This is further validated by the emergence of UV‐Vis characteristics and their subsequent temporal redshift [34]. Based on the integrated results of in situ UV–vis and PL spectroscopy during thermal annealing, the crystallization process can be decomposed into two stages. The first stage corresponds to rapid crystallization, as evidenced by the sharp increase in intensity across both UV–Vis and PL spectra. A marked temporal discrepancy is observed between the pristine and treated films. Specifically, the pristine film exhibits a rise‐and‐fall PL intensity evolution within 8 s. In contrast, the rise‐and‐fall stage for the MACl‐IPA‐treated film is completed within 30 s. The initial enhancement in PL intensity indicates rapid perovskite crystallization, while the subsequent decline results from solvent evaporation and the emergence of grain boundaries during the transition from a wet film to a solid film [35]. Upon further thermal annealing in the second stage, the MACl‐IPA‐treated film enters the recrystallization process. Between 30 and 80 s, a secondary increase in PL intensity is observed for the treated film, indicating the occurrence of recrystallization [36]. The coalescence of small grains and the reduction in overall grain boundary density lead to this secondary enhancement in PL intensity. Simultaneously, the absorption intensity of the film also exhibits a gradual upward trend, indicating the crystallization enhancement. These results demonstrate that the MACl‐IPA treatment effectively regulates the crystallization process of the halide perovskite. By modulating the kinetics toward rapid nucleation and slow growth, a progressive and uniform crystal growth process is promoted, ultimately resulting in larger grain sizes, and enhanced XRD intensities [37].

### 2.2 Halide Perovskite Synapses Performance

GAMA_5_Pb_5_I_16_ halide perovskite synapses can be fabricated using a simple 2T structure as illustrated in Figure 2a, following spin‐coating and thermal evaporation steps (Experimental Section). The Au top electrode, the 2D perovskite active layer, and the ITO bottom electrode emulate the presynaptic membrane, the synaptic cleft, and the postsynaptic membrane, respectively. By tuning the parameters of the input voltage pulses, GAMA_5_Pb_5_I_16_‐based synaptic devices successfully emulated various forms of synaptic plasticity. The tunability of synaptic weight in neuromorphic computing was investigated through the continuous current‐voltage (*I*–*V*) scan. As shown in Figure 2b, the devices exhibit typical analog switching characteristics. During the first five forward scans (0–1 V), the device current increases with successive sweeps, representing a gradual transition from the high‐resistance state (HRS) to the low‐resistance state (LRS), which mimics synaptic potentiation. Conversely, during the subsequent five reverse scans (0–1 V), the current decreases continuously, corresponding to the depression process from LRS back to HRS. The asymmetry of the *I*–*V* curves is primarily attributed to the substantial work function difference between the Au top electrode and the ITO bottom electrode, which forms asymmetric Schottky barriers at the two interfaces [38]. Additionally, we measured the I‐V curves at different voltage sweep rates. As shown in Figure S6, the hysteresis behavior increases as the scanning rate decreases. This is attributed to the fact that the mobility of charged defect ions is lower than that of charge carriers such as electrons or holes. Consequently, ion migration becomes more pronounced at slower scanning rates, underscoring ion migration as the primary mechanism underlying the resistive switching behavior [13]. This reversible and non‐abrupt conductance modulation provides a solid foundation for simulating the dynamic updates of biological synaptic weights.

**FIGURE 2:**
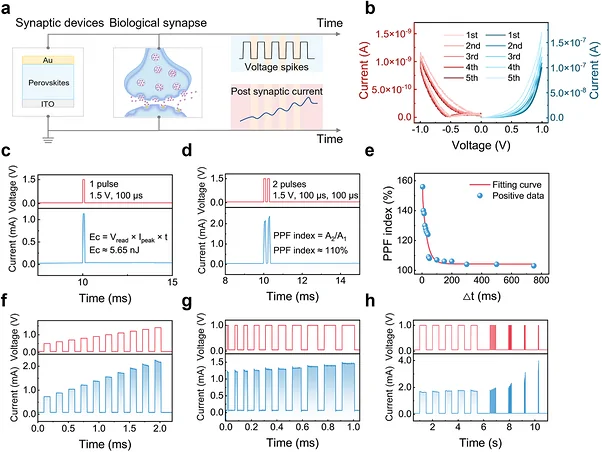
Resistive switching and synaptic plasticity of the perovskite synapse. (a) Schematic illustration of biological and perovskite synapses. (b) Gradual conductance modulation under continuous DC voltage sweeps. (c) Current‐time (*I*–*t*) curves under an electrical pulse with 1.5 V intensity and 100 µs duration. (d) PPF characteristics of the artificial synapse. (e) PPF index as a function of the inter‐pulse interval. (f–h) Synaptic plasticity demonstrating (f) SVDP, (g) SDDP, and (h) SRDP behaviors.

The synaptic plasticity of GAMA_5_Pb_5_I_16_‐based synaptic devices was further investigated under voltage pulse stimulation. The voltage‐stimulated conductance serves as the excitatory postsynaptic current (EPSC), representing the synaptic weight in neuromorphic computing. GAMA_5_Pb_5_I_16_‐based synaptic devices exhibited reconfigurable synaptic responses. As shown in Figure 2c, upon applying a voltage pulse with an intensity of 1.5 V, duration of 100 µs (read at 0.05 V), the device exhibits EPSC behavior. This can be effectively erased through a reverse pulse (Figure S7). The energy consumption was calculated using the product of the voltage (V), current (I), and the duration of the presynaptic pulse (T). For the 1.5 V, 100 µs programming operation, the energy consumption is 169.5 nJ. For the 0.05 V, 100 µs read operation, the synaptic energy consumption is 5.65 nJ. Remarkably, under the 0.05 V, 100 ns read operation, GAMA_5_Pb_5_I_16_‐based synaptic devices demonstrated an ultra‐low energy consumption of 5.5 aJ as shown in Figure S8, which is several orders of magnitude lower than the ∼10 fJ energy consumption of typical biological synapses [39]. In addition, Table S1 lists the performance of representative halide perovskite synapses of the same type, and the perovskite synapse designed in this work achieves the lowest energy consumption. The paired‐pulse facilitation (PPF) behavior of biological synapses was also successfully emulated. As shown in Figure 2d, two consecutive pulses (intensity of 1.5 V, duration of 100 µs, time interval of 100 µs) were applied as presynaptic stimuli, effectively triggering corresponding EPSC responses. The peak EPSC induced by the second pulse is significantly higher than that of the first, exhibiting typical PPF behavior. The PPF index, defined as the ratio of the second peak current (A_2_) to the first (A_1_), was calculated to be 110%. Conversely, paired‐pulse depression (PPD) was observed when applying two consecutive negative pulses (Figure S9), where the second EPSC peak was lower than the first. The PPF index is sensitive to the time interval between pulses (∆t). Using two consecutive pulses (intensity of 1.5 V, duration of 5 ms), as shown in Figure 2e, the PPF index decreases as ∆t increases. This trend follows a double‐exponential function [40, 41]: $\mathrm{PPF}=C_{0}+C_{1}\exp\left(-\frac{\Delta t}{\tau_{1}}\right)+C_{2}\exp\left(-\frac{\Delta t}{\tau_{2}}\right)$, where C_0_ represents a constant, C_1_, and C_2_ indicate the initial magnitudes of facilitation, while τ_1_ and τ_2_ represent the characteristic relaxation times for the fast and slow decay phases, respectively. The fitted τ_1_ and τ_2_ were 5 and 30 ms respectively. The τ_1_ is shorter than τ_2_, and τ_2_ is an order of magnitude larger than τ_1_, which is consistent with the decay kinetics observed in biological synapses [42].

GAMA_5_Pb_5_I_16_‐based synaptic devices also demonstrate spike‐voltage‐dependent plasticity (SVDP), spike‐duration‐dependent plasticity (SDDP), and spike‐rate‐dependent plasticity (SRDP). As shown in Figure 2f,g, synaptic weights increase significantly with higher pulse intensity and longer duration. Furthermore, the device exhibits SRDP behavior (Figure 2h). Under a fixed pulse amplitude, five pulses with different frequencies were applied. We observed that low‐frequency stimuli resulted in minimal weight enhancement, whereas high‐frequency stimuli induced a strong cumulative enhancement of the EPSC. This high‐conductance state at high frequencies is consistent with the PPF effect, which strengthens as the inter‐pulse interval shortens. These results effectively recapitulate the phenomenon of synaptic plasticity in biological neural networks, where frequent, continuous, and more intense stimuli lead to an enhancement of the connectivity between neurons.

Long‐term plasticity is a critical function for biomimetic synaptic devices. Emulating long‐term plasticity enables permanent reconfiguration of neural networks, providing the brain with massive information storage and memory capabilities [52]. This property plays an indispensable role in pattern recognition and information processing tasks within artificial neural networks [53]. To induce long‐term potentiation (LTP) and long‐term depression (LTD), a pulse sequence consisting of 50 positive pulses with an intensity of 1.5 V and 50 negative pulses with an intensity of −1.5 V was applied to the device. The pulse duration was equal to the interval time and was increased stepwise from 100 to 500 µs. Figure 3a shows the extracted LTP and LTD curves. The statistical trends of nonlinearity and asymmetry as a function of pulse duration are presented in Figure 3b, with detailed calculation procedures described in Supporting Information. Specifically, when pulses with a duration and interval of 300 µs were applied, the α_p_ and α_d_ were 0.047 and 0.046, with an asymmetry of only 0.001, as shown in Figure 3e. We also investigated the effect of pulse intensity, as shown in Figure 3c. A pulse sequence consisting of 50 positive pulses and 50 negative pulses was applied, with the absolute pulse intensity increasing from 1.1 to 1.5 V. The statistical trends of nonlinearity and asymmetry relative to the pulse voltage are shown in Figure 3d. The best LTP and LTD performance was achieved at intensities of ±1.1 V, where the α_p_ and α_d_ were 0.010 and 0.012, respectively, with an asymmetry of only 0.002, as shown in Figure 3f. The electrical characteristics of the pristine device under the corresponding operating conditions, including energy consumption and LTP/LTD conductance modulation, are provided in Figure S10. Furthermore, as shown in Figure 3g, the evolution of the dynamic range (the ratio of G_max_ to G_min_) under varying pulse durations and intensities was investigated, both exhibiting a general increasing trend. Next, to increase the number of conductive states, a longer sequence of 100 positive pulses and 100 negative pulses (intensity of ±1 V, duration and interval time of 500 µs) was applied. As shown in Figure 3h, the device successfully achieved 100 continuous conductance states. Notably, the device demonstrated excellent linearity and symmetry, with the α_p_ and α_d_ as low as 0.027 and 0.064, respectively, and an asymmetry of 0.037. Comparative analysis with previous studies reveals that our devices offer a significant advancement in the number of intermediate states while concurrently suppressing nonlinearity, as shown in Figure 3i and Table S1 [13, 22, 24, 43, 44, 45, 46, 47, 48, 49, 50, 51]. Such highly linear and symmetric conductance updates validate the exceptional performance of GAMA_5_Pb_5_I_16_‐based synaptic devices, underscoring its potential for neuromorphic computing applications.

**FIGURE 3:**
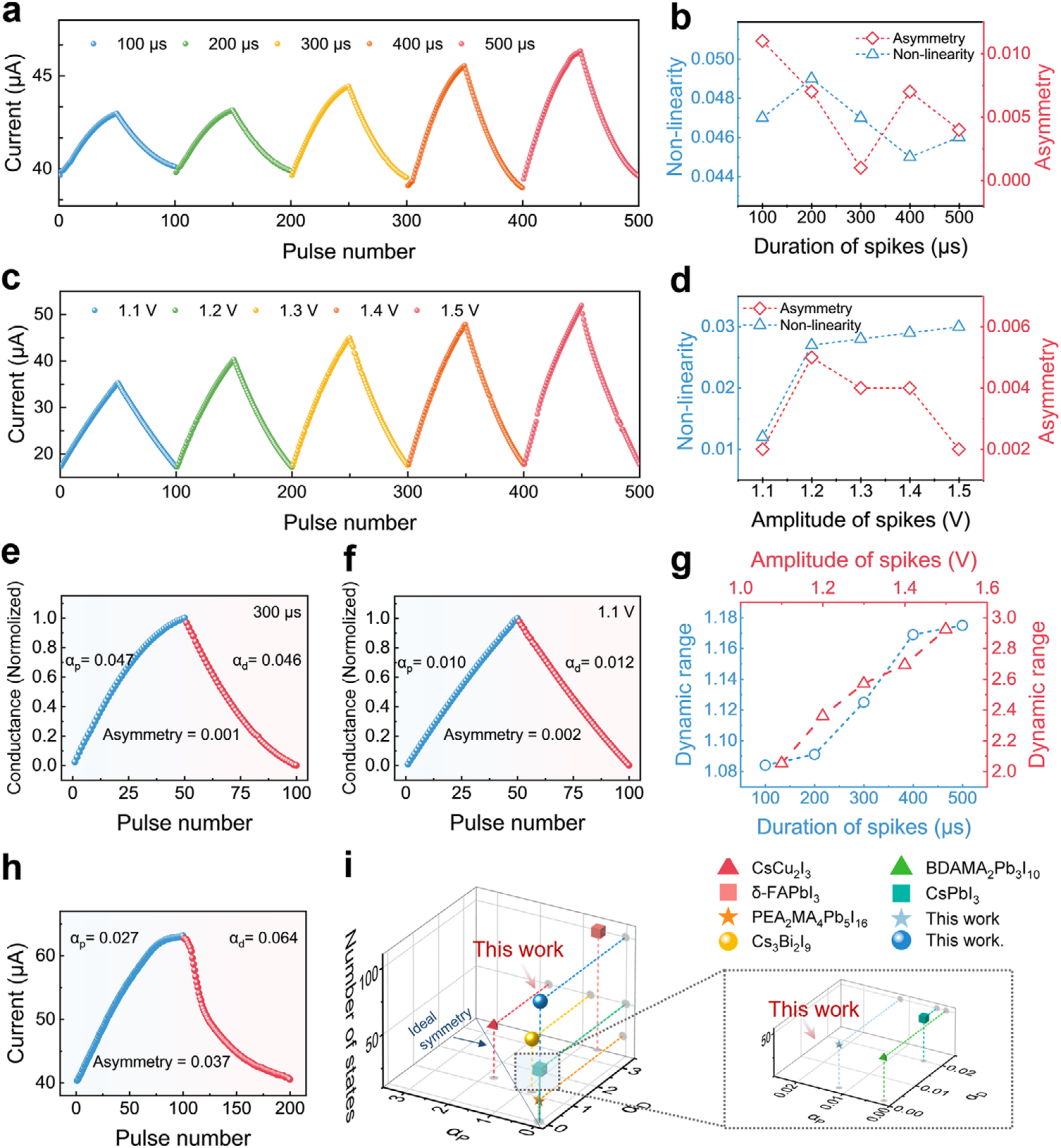
Long‐term plasticity and weight update characteristics. (a) LTP and LTD characteristics of the artificial synapse under pulses with different durations. (b) Statistical trends of nonlinearity and asymmetry as a function of pulse duration. (c) LTP and LTD characteristics under pulses with different intensities. (d) Statistical trends of nonlinearity and asymmetry as a function of pulse intensity. (e) LTP and LTD curves showing 50 continuous conductance states under pulses with a duration of 300 µs. (f) LTP and LTD curves showing 50 continuous conductance states under pulses with a 1.1 V intensity. (g) Evolutionary trend of the dynamic range as a function of pulse duration and pulse intensity. (h) LTP and LTD curves showing 100 continuous conductance states under pulses with a 1 V intensity. (i) Comparison of the nonlinearity as well as number of states and amplification diagrams of various 2T halide perovskite artificial synapses [13, 24, 43, 44, 45, 46, 47, 48, 49, 50, 51].

We further explored the dominant mechanism of GAMA_5_Pb_5_I_16_‐based synaptic devices. Given the inert Au electrodes and the incorporated PMMA insulating layer, the formation of metallic conductive filaments through potential reactions with the electrodes is unlikely. It is generally accepted that the dominant mechanism is a valence change mechanism (VCM). As typical mixed ionic‐electronic conductors, all ions and their vacancies at the A, B, and X sites in halide perovskites can participate in multi‐state conductance operations. Due to their relatively low activation energy for migration, iodine ions (I^−^) and iodine vacancies (V_I_) are generally considered the primary ions responsible for the conductance changes [54].In addition, density functional theory calculations confirm that iodine‐vacancy migration is favored along the inorganic Pb─I framework and suppressed across the organic spacer, with vacancies preferentially formed within the inorganic framework, accounting for the confined, and reproducible conductance updates (Figure S13). Furthermore, to identify the specific category of the VCM mechanism, we investigated the *I*–*V* characteristics across different electrode areas. As described in Figures S11 and S12, the device current exhibits a clear trend of increasing with the effective area. This characteristic indicates that charged ions migrate uniformly across the entire effective electrode interface. This aligns with the features of an interfacial‐type valence change mechanism rather than a localized conductive filament mechanism [14]. These results demonstrate that the analog resistive switching behavior originates from the uniform modulation of the interface charge distribution. Based on this analysis, we divided the forward *I*–*V* characteristic curve into four parts to explain the working mechanism in more detail (Figure S14).

To validate GAMA_5_Pb_5_I_16_‐based synaptic devices for neuromorphic vision computing architectures, fundamental image recognition tasks were first evaluated. A three‐layer artificial neural network (ANN) was designed to implement handwritten digit recognition using the Modified National Institute of Standards and Technology (MNIST) database as shown in Figure S15. Each image in the dataset has a resolution of 28 × 28 pixels. A total of 60 000 samples were selected, with 80% used for training and the remaining 20% used for testing. The ANN architecture consists of an input layer with 784 neurons and an output layer with 10 neurons, representing digits 0 through 9. These layers are interconnected via a 784 × 100 synaptic weight matrix and a 100 × 10 synaptic weight matrix. Detailed information regarding the simulation process is provided in the Experimental Section. The recognition accuracy increased to 93.2% after 30 training epochs, demonstrating high performance in pattern recognition. The confusion matrix shows that most digits were accurately classified. These results demonstrate that ACI‐phase perovskite‐based synaptic devices with high linearity and symmetry, enable precise adjustment of analog synaptic weights by modulating conductance, thus offering new possibilities for future development of neuromorphic hardware.

### 2.3 Implementations of Object Detection

Based on the obtained parameters of the GAMA_5_Pb_5_I_16_‐based synaptic devices, a simulated neuromorphic computing system (PS‐YOLOv9) is constructed to perform complex visual tasks, including object detection, image segmentation, and perception‐driven classification. Inspired by the human visual system, as shown in Figure 4a, the presented system integrates the advanced single‐stage YOLO v9 algorithm and utilizes the conductance potentiation and depression characteristics of the perovskite synaptic devices to achieve device‐constrained weight projection within the software simulation framework. For evaluation, we employed the A2D2 (Audi Autonomous Driving Dataset) [55], which covers five core traffic elements: vehicles, pedestrians, traffic lights, traffic signs, and obstacles.

**FIGURE 4:**
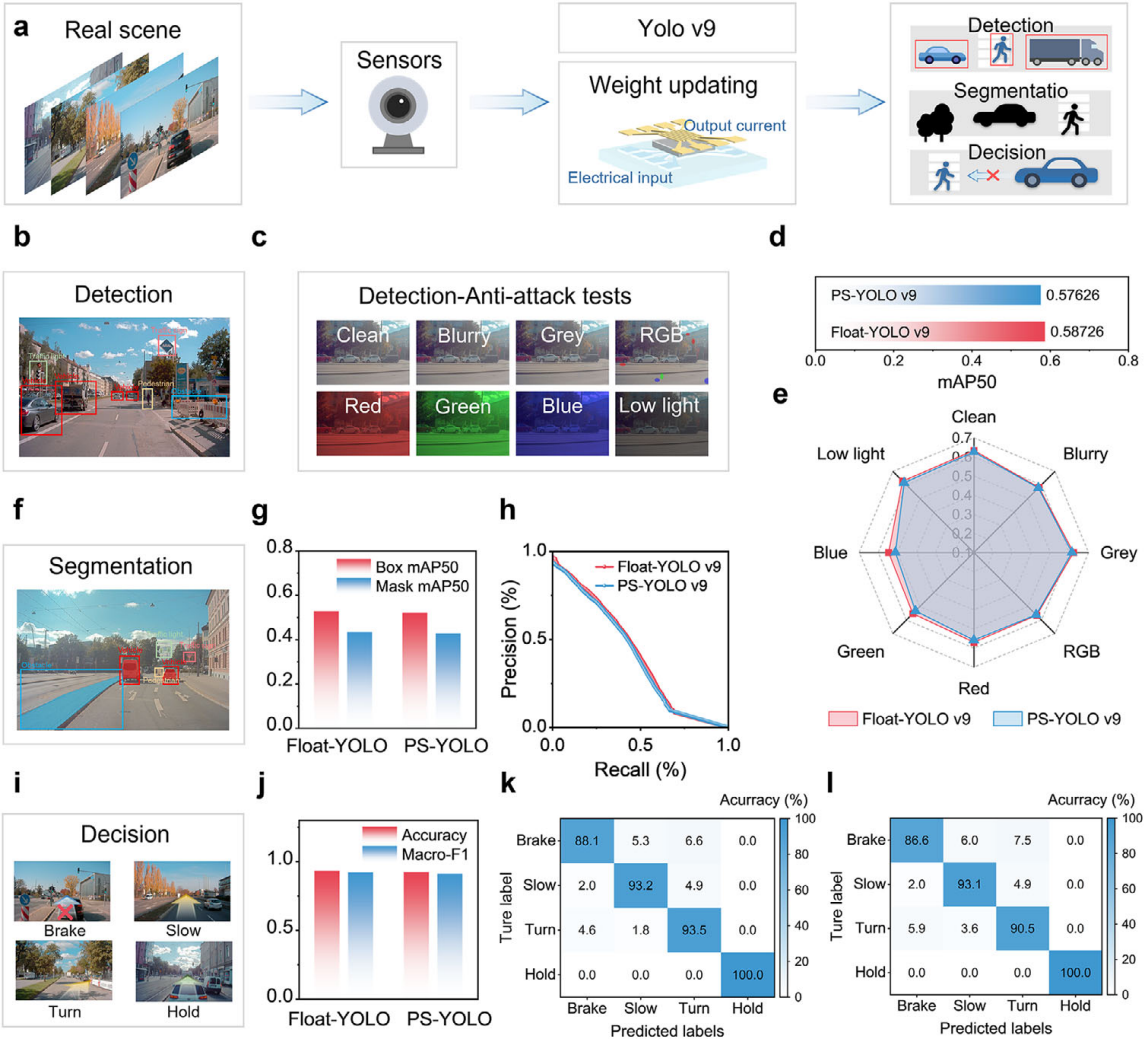
Detection, segmentation, and decision‐making enabled by perovskite synapses. (a) Schematic illustration of the PS‐YOLOv9 on the A2D2 dataset. (b) Schematic of the object detection task. (c) Example images under the clean baseline and seven visual perturbations. (d) Comparison of detection mAP50 for the float baseline and PS‐YOLOv9. (e) Detection robustness under the clean baseline and seven perturbations. (f) Schematic of the semantic segmentation task. (g) Box and Mask mAP50 for the float baseline and PS‐YOLO v9. (h) Precision‐Recall (PR) curves for the segmentation task. (i) Schematic of the decision‐making process. (j) Comparison of Accuracy and Macro‐F1 for the float baseline and PS‐YOLO v9. (k, l) Confusion matrices of decision classification results for (k) the float baseline and (l) PS‐YOLO v9. Street‐view images in panels (a–c, f, i) are adapted from the A2D2: Audi Autonomous Driving Dataset (https://www.a2d2.audi), Geyer et al., arXiv:2004.06320 (2020), released under the CC BY‐ND 4.0 license.

For the object detection task (Figure 4b), we evaluated the detection precision of the dataset after training, and compared the performance of PS‐YOLO v9 with the floating‐point baseline (Float‐YOLO v9). The mean Average Precision at an intersection over union (IoU) threshold of 0.5 (mAP50) was used as the core evaluation metric. As shown in Figure 4d, PS‐YOLO v9 achieved an mAP50 of 57.6%, approaching the 58.7% of Float‐YOLO v9. The absolute mAP50 gap between the two is only 1.1%, and the relative performance retention is as high as 98.1%. This indicates that under the same training framework, the precision loss introduced by memristor‐aware weight projection is minimal. Furthermore, to verify the model's robustness, we performed visual attack tests on the dataset images consisting of a clean baseline and seven distinct perturbations. These perturbations included blurry, gray patches, RGB patches, red tints, green tints, blue tints, and low‐light conditions (Figure 4c). The results for mAP50 across these seven attack modes are shown in Figure 4e. Although the PS‐YOLO v9 model performs slightly worse than the Float‐YOLO v9 model under these disturbances, the degradation is minor, and the model remains functional under all conditions.

In addition to object detection, the PS‐YOLO v9 model successfully executed image segmentation tasks, as illustrated in Figure 4f. Box mAP50 and Mask mAP50 were used to evaluate target localization accuracy and pixel‐level mask generation overlap, respectively. Box mAP50 measures the IoU between the predicted and ground‐truth boxes (coarse‐grained localization), while Mask mAP50 measures the pixel‐level overlap between predicted and ground‐truth masks. The PS‐YOLO v9 model demonstrated excellent segmentation performance, achieving accurate recognition, classification, and mask generation for various objects. As shown in Figure 4g, the Box and Mask mAP50 values for the Float‐YOLO v9 model were 52.8% and 43.5%, respectively, while those for the PS‐YOLO v9 model were 52.1% and 42.8%, respectively. The relative differences for Box and Mask mAP50 were approximately 1.42% and 1.70%. The device‐constrained model maintains exceptional performance, with relative retention rates of 98.6% for the bounding box and 98.3% for the mask mAP. We further evaluated the precision and recall trade‐offs using Precision‐Recall (PR) curves across different decision thresholds. As shown in Figure 4h, the curves for both models match closely in the segmentation task, further confirming that the perovskite synaptic devices provide reliable support for high‐complexity visual perception tasks.

Finally, we investigated the decision‐making function of PS‐YOLO v9 model, as shown in Figure 4i. The classification performance was comprehensively measured using accuracy and the Macro‐F1 score. Accuracy reflects the overall proportion of correct predictions across four decision categories: Brake, Slow, Turn, and Hold. The Macro‐F1 score is calculated by determining the F1 score (the harmonic mean of precision and recall) for each of the four categories and then averaging them, reflecting the balance of the model in learning each driving behavior. As shown in Figure 4j, the Accuracy and Macro‐F1 scores for the Float‐YOLO v9 model were 93.1% and 92.1%, respectively, while the PS‐YOLO v9 model achieved 92.3% and 91.1%, respectively. The absolute differences for both metrics are within approximately 1%. The relative performance retention rates for accuracy and Macro‐F1 score reach as high as 99.1% and 98.9%, respectively. The confusion matrices for both models (Figure 4k,l) clearly demonstrate high prediction rates for all driving decisions, highlighting the remarkable performance of the object detection system based on halide perovskite synapses.

### 2.4 Image Memory With Halide Perovskite Synaptic Arrays

Beyond emulating basic neural behaviors, integrating synaptic devices into large‐scale arrays is a core prerequisite for building brain‐like computing systems [56]. To verify the potential of GAMA_5_Pb_5_I_16_‐based synaptic devices for high‐performance parallel computing and distributed information storage, an 8 × 8 crossbar array was fabricated as shown in Figure 5a–c. To evaluate the hardware reliability of the fabricated array, the I‐V properties of all 64 memristive units were examined. All units demonstrated effective resistive switching under continuous voltage sweeps from −1 to 1 V (Figure S16), confirming a high device yield. Furthermore, to demonstrate the weight storage capability, we simulated image memory applications by programming a Z‐shaped pattern into the 8 × 8 array. The array successfully demonstrates both volatile short‐term and stable long‐term image memory functions by modulating pulse durations to simulate the transition from rapid perception to long‐term information retention. Figure S17 maps the spatial distribution and the number of programming pulses, where each pulse had an intensity of 1.5 V and a duration of 100 ns. As shown in Figure 5d–f, the initial background current of the array was low. Upon applying the electrical stimulation, the array current responded rapidly and reached its peak, displaying eight distinguishable conductance levels corresponding to the pulse counts. Due to the volatile nature of the devices, the current decayed to its initial level within only 1 s after the pulses were removed. This process successfully emulates the rapid perception and forgetting process of the biological visual system when handling transient environmental information. Subsequently, to induce the long‐term memory process, the pulse duration was extended to 500 µs. As shown in Figure 5g–i, extending the pulse duration allows the programmed Z‐shaped pattern to persist for over 30 s.

**FIGURE 5:**
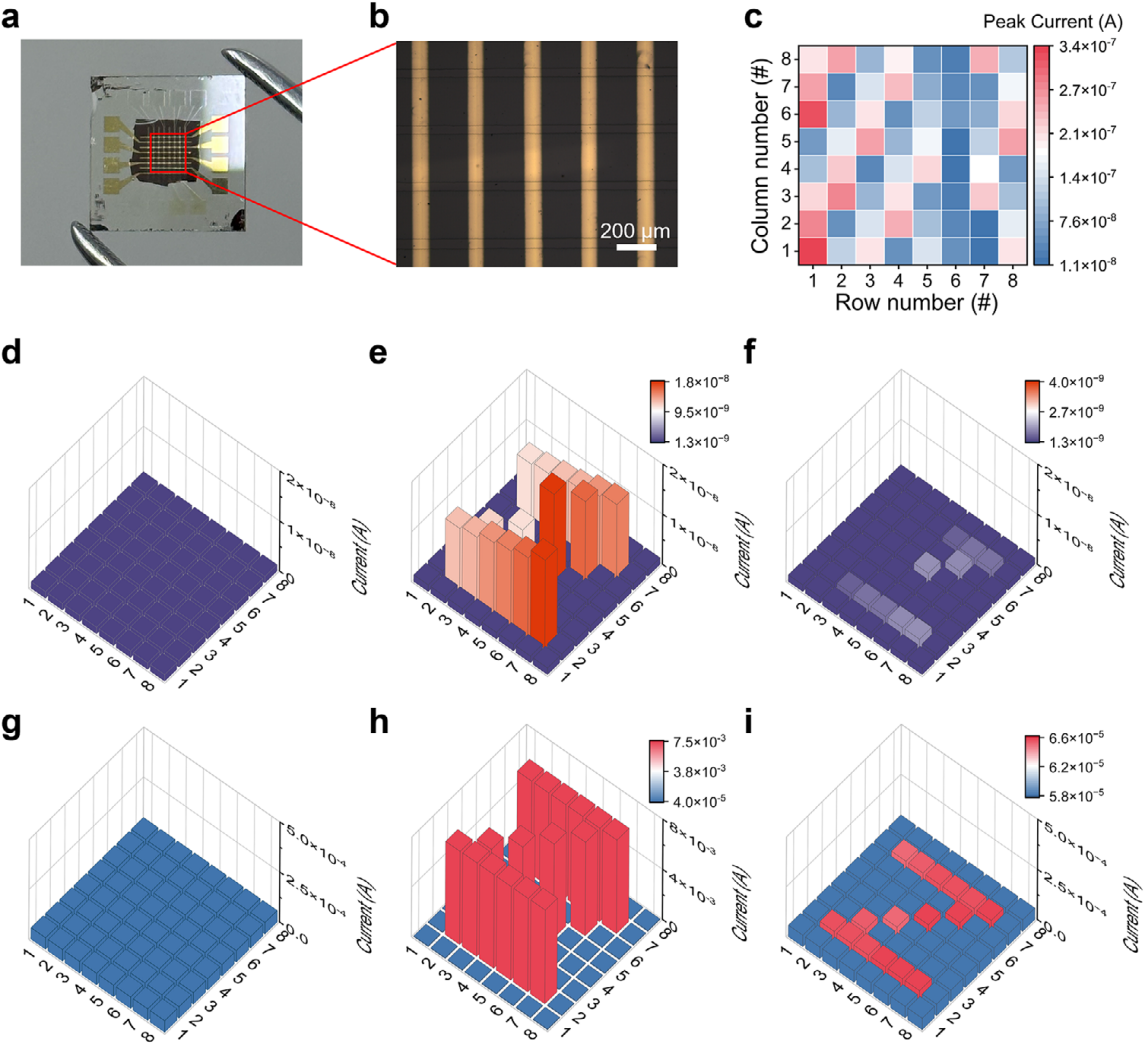
Image memory simulation in the 8 × 8 perovskite synaptic array. (a) Image of the patterned ITO substrate. (b) Image and (c) magnified light microscope image of the halide perovskite synaptic array. (d) Initial background current distribution before programming during short‐term memory simulation. (e) Transient peak current response after applying pulses with a 1.5 V intensity and a 100 ns duration. (f) Spatial current distribution 1 s after the pulses ended. (g) Initial background current distribution for the long‐term memory simulation. (h) Transient current response after applying pulses with a 1.5 V intensity and a 500 µs duration. (i) Spatial current distribution 30 s after the pulses ended.

## 3 Conclusion

In summary, we demonstrate a multistate and linearly programmable artificial synapse based on GAMA_5_Pb_5_I_16_ halide perovskite, fabricated via a synergistic crystallization modulation technique. The resulting vertical crystal orientation facilitates controllable ion migration and efficient carrier transport, underpinning the device's highly linear and symmetric conductance modulation. The fabricated device achieves a minimum energy consumption of 5.5 aJ per synaptic event. For 50 conductance states, the non‐linearity is only 0.010 and 0.012 with an asymmetry of 0.002. Remarkably, even when scaled to 100 conductance states, the non‐linearity remains as low as 0.027 and 0.064 with an asymmetry of 0.037. Furthermore, an 8×8 crossbar synaptic array was successfully fabricated with high yield. Moreover, high‐accuracy MNIST recognition and object detection have been successfully demonstrated via system‐level simulations based on the halide perovskite synapses. These exceptional capabilities demonstrate the significant potential of halide perovskite synapses in machine vision tasks, providing a strong foundation for their future integration into neuromorphic computing systems in intelligent transportation and autonomous driving.

## 4 Experimental Section

### 4.1 Materials

Guanidinium iodide (GAI, 99.5%) and methylammonium iodide (MAI, 99.9%) were purchased from Xian Yuri Solar Co., Ltd. Lead iodide (PbI_2_, 99.99%) and methylammonium chloride (MACl, 99.9%) were obtained from TCI Company. PMMA (acrylic, Mw ∼350 000), anhydrous N,N‐dimethylformamide (DMF, 99.8%), anhydrous dimethyl sulfoxide (DMSO, 99.8%), and anhydrous chlorobenzene (CB, 99.8%) were purchased from Sigma–Aldrich. Ethyl acetate (EA, ≥99.8%) was obtained from Meryer. All powders and solvents were used as received without further purification.

### 4.2 Device Fabrication

Indium tin oxide (ITO) coated glass substrates (1.5 cm × 1.5 cm, 1 mm thick, Liaoning Youxuan New Energy Technology Co., Ltd.) were sequentially cleaned via ultrasonication in deionized water, ethanol, acetone, and isopropanol (IPA) for 5 min each. The substrates were then treated with ultraviolet‐ozone (UV‐ozone) treatment for 20 min, before transferred to a glove box. The perovskite precursor solution (1.2 m) was prepared by dissolving GAI, MAI, and PbI_2_ (molar ratio 1:5:5) with 10 mg/mL MACl in a mixed solvent of DMF and DMSO (19:1 by volume). The solution was stirred thoroughly and filtered through a 0.22 µm PTFE syringe filter. For the PMMA solution, 10 mg of PMMA was dissolved in 2 mL of chlorobenzene (CB) and stirred at 70°C for 6 h to achieve a concentration of 5 mg/mL. Using a one‐step anti‐solvent method, the precursor was spin‐coated at 1000 rpm for 3 s, followed by 3000 rpm for 20 s. During the last 10 s of the second stage, 450 µL of ethyl acetate was dropped to accelerate crystallization. Subsequently, the films were annealed at 100°C for 20 min using an IPA solvent‐thermal annealing method. Next, the PMMA solution was spin‐coated onto the perovskite film at 5000 rpm for 30 s and annealed at 100°C for 10 min. All procedures were conducted in a nitrogen‐filled glovebox. Finally, an 80‐nm‐thick gold (Au) top electrode was deposited via thermal evaporation.

### 4.3 Array Fabrication

The ITO glass substrates were cleaned and treated using the same protocol described above. The perovskite and PMMA layers were fabricated following the aforementioned methods. Local areas of the perovskite and protective layers were then mechanically removed (scraped) to expose the underlying ITO for bottom electrode contact. Finally, patterned Au electrodes were deposited through a shadow mask via thermal evaporation, forming a crossbar array. Both the top and bottom electrodes have a width of 100 µm, resulting in a device active area of approximately 10 000 µm^2^ (100 µm × 100 µm).

### 4.4 Characterization

The surface and cross‐sectional morphologies of the perovskite films were characterized using scanning electron microscopy (SEM, Hitachi SU8600). The surface roughness was analyzed via atomic force microscopy (AFM, Bruker Dimension Icon). Ultraviolet‐visible (UV–vis) absorption spectra and X‐ray photoelectron spectroscopy (XPS) were measured using a Shimadzu UV‐3600i PLUS spectrophotometer and a Thermo Scientific ESCALAB 250Xi system, respectively, to investigate the optical properties and chemical compositions of the films. Steady‐state photoluminescence (PL) and time‐resolved photoluminescence (TRPL) spectra were obtained using a fluorescence spectrometer (Edinburgh FLS920). X‐ray diffraction (XRD) data were acquired at room temperature using a Rigaku‐2038 diffractometer. Grazing‐incidence wide‐angle X‐ray scattering (GIWAXS) data were acquired at the 1W1A Diffuse X‐ray Scattering Station, Beijing Synchrotron Radiation Facility (BSRF). The resistive switching characteristics and synaptic plasticity of the devices were measured using Semiconductor Device Parameter Analyzer equipped with a waveform generator/fast measurement unit (WGFMU) module. During all electrical measurements, the bottom electrode was grounded. All characterizations and tests were performed at room temperature under ambient conditions.

### 4.5 Pattern Recognition Simulation

For MNIST handwritten digit recognition, we implemented a multilayer perceptron (MLP) architecture. The network consists of an input layer with 784 neurons, a hidden layer with 100 fully connected neurons, and an output layer with 10 neurons. These layers are interconnected through two synaptic weight matrices of 784 × 100 and 100 × 10, respectively. The training utilized a dataset of 60 000 images, processed with a mini‐batch size of 64. This configuration resulted in approximately 938 iterations per epoch. To integrate the hardware characteristics into the learning framework, the synaptic weights were mapped to the experimentally measured conductance states from the LTP and LTD curves. During the weight update process, the algorithm determined the required weight change based on the calculated gradient and searched for the nearest available conductance value on the corresponding LTP or LTD curve. A write‐verify scheme was employed to ensure the accuracy of the conductance states and to confirm successful transitions. A constant learning rate of 0.001 was applied throughout the training. The activation of each neuron was transmitted to the subsequent layer through the synaptic weights and processed by a Sigmoid activation function. Python was used to implement the pattern recognition processing described above.

### 4.6 Detection, Segmentation, and Decision‐Making Simulation

All experiments were conducted in a Python/PyTorch software environment on a single NVIDIA RTX 5090 GPU. Object detection and instance segmentation were implemented using YOLOv9‐C and GELAN‐C‐SEG, respectively, on the A2D2 dataset with five classes [55]. The floating‐point detection model was trained for 100 epochs (batch size 4, input size 640), and the memristor‐aware detector was initialized from the best checkpoint and trained for 60 more epochs (batch size 4). For segmentation, the floating‐point model was trained for 60 epochs (batch size 8, input size 512), and the memristor‐aware model was then trained for 40 additional epochs (batch size 8). AdamW and cosine learning‐rate decay were used. The decision module used a multilayer perceptron (hidden dimension 64) trained for 200 epochs.

### 4.7 Memristor‐Aware Weight Projection

The memristor‐aware results were obtained through software simulation, not direct measurements from a fabricated crossbar array. Experimentally measured LTP/LTD conductance‐update curves were converted into normalized, nonuniform conductance‐state tables (100 states for detection, 50 for segmentation). Signed weights used a differential scheme; eligible convolutional weights were clipped to |w| ≤ 0.25 and projected to the nearest conductance state, with a straight‐through estimator for backpropagation. The detection and segmentation heads were retained in floating point, while eligible backbone and neck layers were projected. Under this configuration, the floating‐point and memristor‐aware detection models achieved mAP50 values of 0.58726 and 0.57626, respectively.

### 4.8 Scope of Modeled Device Characteristics

The present model includes two measured device characteristics: (i) the finite number of conductance states and (ii) the nonuniform spacing between adjacent conductance states, both extracted from the measured LTP/LTD curves of a single device. Device‐to‐device (cell‐to‐cell) variation and cycle‐to‐cycle variation were set to 0, and endurance degradation and retention drift were not included. Accordingly, the reported performance represents software‐simulation results under nominal, measured‐curve‐informed conductance constraints, and should not be interpreted as direct hardware or long‐term operational accuracy.

### 4.9 Computational Details

All calculations were based on density functional theory (DFT) as implemented in the Vienna Ab initio Simulation Package (VASP) [57, 58]. The electron‐ion interactions were described by the projector augmented‐wave (PAW) method [59]. The exchange‐correlation functional was treated within the generalized gradient approximation (GGA) [60] parameterized by Perdew, Burke, and Ernzerhof (PBE) [61]. A plane‐wave cutoff energy of 600 eV was employed. The Brillouin zone was sampled using a Monkhorst‐Pack k‐point mesh of 3 × 3 × 1 for structural relaxations. During structural relaxation, the bottom layers were fixed, while the other layers along with the adsorbates were allowed to relax fully. The electronic self‐consistent cycle was considered converged when the total energy change between successive steps was less than 1 × 10^−5 ^eV. Geometry optimization was performed until the Hellmann‐Feynman forces on all atoms were below 0.02 eV/Å. The minimum number of electronic steps was set to 6, and the maximum number was set to 200. The maximum number of ionic steps was set to 300. D3 correction was used to correct for the long‐range forces [62]. A dipole correction was applied along the vacuum direction.

## Author Contributions

Z.S., B.W., and H.S. designed the experiments. Z.S., B.W., Z.Z., and Z.W. performed the experiments related to material preparation and performance testing. Z.S. and Z.W. fabricated the devices, performed the measurements, and analyzed the electrical characteristics. Z.S. carried out the simulation experiments on image recognition and object detection. Z.S. drafted the original manuscript. X.Z., H.S., Q.L., and Y.Z. supervised and administered the project. All authors joined the discussions and revised the manuscript.

## Conflicts of Interest

The authors declare no conflicts of interest.

## Supporting information




**Supporting File**: advs78461‐sup‐0001‐SuppMat.docx

## Data Availability

The data that support the findings of this study are available from the corresponding author upon reasonable request.
