# Supplementary material for: Highly Linear Perovskite Synapses for Neuromorphic Computing
Source: Adv Sci (Weinh). 2026 Oct 26:e78461. Online ahead of print. doi: 10.1002/advs.78461 (PMC13710706; doi:10.1002/advs.78461)
Supplement: Supplementary file 1 [file ADVS-9999-e78461-s001.docx]

Supporting Information

**Highly Linear Perovskite Synapses for Neuromorphic Computing**

*Zhongwen Sun^1,2^, Boyu Wan^1,2^, Zifan Zhang^1,2^, Zhikai Wang^1,2^, Xuan Zhao^1,2^, Haonan Si^1,2,*^, Qingliang Liao^1,2,*^, Yue Zhang^1,2,*^*

^1^ Academy for Advanced Interdisciplinary Science and Technology, Key Laboratory of Advanced Materials and Devices for Post-Moore Chips Ministry of Education, Beijing Key Laboratory for Advanced Energy Materials and Technologies, University of Science and Technology Beijing, Beijing 100083, P. R. China.

^2^ School of Materials Science and Engineering, State Key Laboratory for Advanced Metals and Materials, University of Science and Technology Beijing, Beijing 100083, P. R. China.

Correspondence: Haonan Si ([sihaonan@ustb.edu.cn](mailto:sihaonan@ustb.edu.cn)); Qingliang Liao ([liao@ustb.edu.cn](mailto:liao@ustb.edu.cn)); Yue Zhang ([yuezhang@ustb.edu.cn](mailto:yuezhang@ustb.edu.cn))

Figure S1. Atomic force microscopy (AFM) images of the (a) pristine and (b) MACl-IPA treated films.

**Figure S2.** (a) Cross-sectional SEM image of the 2D perovskite film with MASCN additive. Elemental mapping of (b) In, (c) I, and (d) Pb.

**Figure S3**. Steady-state photoluminescence (PL) spectra of the (a) pristine and (b) MACl-IPA treated film.

Figure S4. (a) UV-Vis absorption spectra and (b) corresponding Tauc plot of the MACl-IPA treated perovskite film.

Figure S5. X-ray photoelectron spectroscopy (XPS) analysis of the perovskite films before and after MACl-IPA treatment. (a) XPS survey spectra. High-resolution XPS spectra of (b) Cl 2p, (c) Pb 4f, and (d) I 3d orbitals.

**Figure S6.** Sweep-rate-dependent I-V curves in the (a) negative voltage range (0 to -1 V) and (b) positive voltage range (0 to 1 V).

**Figure S7.** (a) Current-time curve under a pulse with a -1.5 V intensity and a 100 us duration. (b) Time-dependent current recorded after pulse erasure.

**Figure S8.** Current-time curve under a pulse with a 1.5 V intensity and a 100 ns duration.

**Figure S9.** (a) PPD characteristics of the artificial synaptic device. (b) PPD index as a function of the inter-pulse interval.

Figure S10. I-V characteristic curves under negative voltage sweeps with electrode size of (a) 100 um, (b) 200 um, (c) 300 um, (d) 400 um, and (e) 500 um. (f) Statistical trend of the peak current as a function of electrode dimension during the scanning process.

Figure S11. I-V characteristic curves under positive voltage sweeps with electrode size of (a) 100 um, (b) 200 um, (c) 300 um, (d) 400 um, and (e) 500 um. (f) Statistical trend of the peak current as a function of electrode dimension during the scanning process.

Figure S12. (a) Forward I-V curve of the perovskite synaptic device. (b) Schematic illustration of internal ion migration within the device. (c, d) Schematic diagrams showing the resistive switching mechanism and the energy band alignment transition at the Schottky interface.

**Figure S13. Handwritten digit recognition applications based on halide perovskite synapses.** (a) Schematic illustration of the digit recognition process in the neural network. (b) LTP and LTD characteristics of the perovskite synaptic device. (c) Evolution of the recognition accuracy over training epochs. (d) Confusion matrix generated at the 30th training epoch.

Figure S14. (a) Logarithmic and (b) linear plots of the I-V characteristic curves for the 64 memristive units in the array.

**Figure S15**. (a) Programming pulse distribution mapping for the Z-shaped pattern during short-term memory simulation with pulses of 1.5 V, 100 ns. (b) Programming pulse distribution mapping for the long-term memory simulation with pulses of 1.5 V, 500 μs.

**Supplementary Note 1: Calculation of Nonlinearity and Asymmetry**

The formula for calculating nonlinearity is as follows

$\text{G}_{\text{p}}\text{=}\text{G}_{\text{min}}\text{+B(1-}\text{e}^{\text{-N/}\text{A}_{\text{p}}}\text{)}$	(1)

$\text{G}_{\text{d}}\text{=}\text{G}_{\text{max}}\text{-B(1-}\text{e}^{\text{(N-}\text{N}_{\text{max}}\text{)/}\text{A}_{\text{d}}}\text{)}$	(2)

$\text{B=}\frac{\text{G}_{\text{max}}\text{-}\text{G}_{\text{min}}}{\text{1-}\text{e}^{\text{-}\text{N}_{\text{max}}\text{/}\text{A}_{\text{p,d}}}}$	(3)

$\text{α}_{\text{p,d}}\text{=}\frac{\text{1.726}}{\text{A}_{\text{p,d}}\text{+0.162}}$	(4)

G_p_ and G_d_ represent the conductance during the potentiation and depression processes, respectively. G_max_, G_min_, and N denote the maximum conductance, minimum conductance, and the number of applied pulses. A_p_ and A_d_ are parameters related to nonlinearity, while B is a factor used to normalize the conductance values. The degree of nonlinearity is represented by$\text{α}$_p_ and $\text{α}$_d_.

The symmetry of the device is quantitatively characterized by the asymmetry value, which is defined as the absolute difference between the potentiation and depression nonlinearity factors:

Asymmetry=||$\text{α}$_p_|-|$\text{α}$_d_||	(5)

**Table S1. Performance comparison of representative halide perovskites synapses**

| Device structure | Energy consumption | Number of states | Nonlinearity | Asymmetry | Ref. |
| --- | --- | --- | --- | --- | --- |
| Au/FAPbI_3_/ITO | - | 20 | - | - | [1] |
| Ag/PMMA/Cs_2_AgBiBr_6_/ITO | - | 50 | - | - | [2] |
| Au/CsCu_2_I_3_/ITO | - | 50 | 1.8/1.3 | 0.5 | [3] |
| Ag/δ-FAPbI_3_/ALD-SnO_2_/ITO | - | 100 | 0.6/3.47 | 2.87 | [4] |
| Au/PMMA/PEA_2_MA_4_Pb_5_I_16_/Au | - | 25 | 0.07/0.1 | 0.03 | [5] |
| Au/PMMA/BDAMA_n-1_Pb_n_I_3n+1_/Au | 2.1 pJ | 50 | 0.002/0.0015 | 0.0005 | [6] |
| Au/PMMA/Cs_3_Bi_2_I_9_/PMMA/Au | - | 50 | 0.825/1.136 | 0.311 | [7] |
| Ag/PMMA/Cs_3_Cu_2_I_5_/ITO | - | 20 | - | - | [8] |
| Au/PMMA/GAMA_5_Pb_5_I_16_/ITO | 5.485 aJ | 50 | 0.01/0.012 | 0.002 | This work |
|  |  | 100 | 0.027/0.064 | 0.037 |  |

**References**

1.	J. Gong, H. Wei, Y. Ni, et al., “Methylammonium halide-doped perovskite artificial synapse for light-assisted environmental perception and learning,” *Materials Today Physics* 21 (2021): <https://doi.org/10.1016/j.mtphys.2021.100540>.

2.	J. Lao, W. Xu, C. Jiang, et al., “An air-stable artificial synapse based on a lead-free double perovskite Cs_2_AgBiBr_6_ film for neuromorphic computing,” *Journal of Materials Chemistry C* 9 (2021): 5706-5712, <https://doi.org/10.1039/d1tc00655j>.

3.	K. J. Kwak, J. H. Baek, D. E. Lee, et al., “Ambient stable all inorganic CsCu_2_I_3_ artificial synapses for neurocomputing,” *Nano Letters* 22 (2022): 6010-6017, <https://doi.org/10.1021/acs.nanolett.2c01272>.

4.	S.-U. Lee, S.-Y. Kim, J.-H. Lee, et al., “Artificial Synapse Based on a δ-FAPbI_3_/atomic-layer-deposited SnO_2_ bilayer memristor,” *Nano Letters* (2024): <https://doi.org/10.1021/acs.nanolett.4c00253>.

5.	S. J. Kim, T. H. Lee, J.-M. Yang, et al., “Vertically aligned two-dimensional halide perovskites for reliably operable artificial synapses,” *Materials Today* 52 (2022): 19-30, <https://doi.org/10.1016/j.mattod.2021.10.035>.

6.	S. J. Kim, I. H. Im, J. H. Baek, et al., “Linearly programmable two-dimensional halide perovskite memristor arrays for neuromorphic computing,” *Nature Nanotechnology* 20 (2024): 83-92, <https://doi.org/10.1038/s41565-024-01790-3>.

7.	S. K. Vishwanath, C. Karkera, T. Mohammad, et al., “Lead-free Cs_3_Bi_2_I_9_ perovskite memristors for energy-efficient neuromorphic computing,” *ACS Energy Letters* 10 (2025): 2193-2202, <https://doi.org/10.1021/acsenergylett.5c00411>.

8.	F. Zeng, Y. Guo, W. Hu, et al., “Opportunity of the lead-free all-inorganic Cs_3_Cu_2_I_5_ Perovskite film for memristor and neuromorphic computing applications,” *ACS Applied Materials & Interfaces* 12 (2020): 23094-23101, <https://doi.org/10.1021/acsami.0c03106>.

**Supplementary Note 2: Mechanisms of Halide Perovskite Synapses**

At lower voltages (less than 0.2 V, Stage 1), ion migration under the electric field is weak. During this stage, charge carriers struggle to overcome the Schottky barrier, resulting in a low device current, as illustrated in **Figure S12a**. As the voltage increases (from 0.2 to 1.0 V, Stage 2), ions migrate and gradually accumulate at the interface between the electrode and the perovskite material. This accumulation modulates the Schottky barrier, allowing carriers to overcome the barrier and leading to a progressive increase in device current. As shown in Figures **S12b and S12d**, during this process, iodide ions (I^-^) and iodine vacancies (V_I_^+^) with low migration activation energies drift under the driven voltage. This lead to n-type and p-type self-doping at the interface, which modulates the interface Schottky barrier and carrier injection, ultimately regulating the conductance levels. In the reverse scan process, the current in Stage 3 is greater than that in Stage 2 due to enhanced carrier injection. Simultaneously, the redistribution of ions within the perovskite forms a built-in electric field (V_built-in_) that opposes the external electric field (V_external_). When the voltage decreases to approximately 0.05 V, a dynamic balance of carrier motion is reached at the boundary between Stage 3 and Stage 4, where the current reaches its minimum value. During Stage 4, as the external voltage continues to decrease, V_external_ becomes smaller than V_built-in_, ions near the interface begin to diffuse due to the concentration gradient, resulting in a subsequent increase in current.

Overall, the interface-type valence change mechanism facilitates the high-performance analog resistive switching of the Au/PMMA/GAMA_5_Pb_5_I_16_/ITO device structure, which stems from the precise control of material orientation. By introducing additives into the ACI-phase perovskite precursor and controlling the annealing atmosphere, the crystallization kinetics are effectively regulated. Consequently, the perovskite grains preferentially grow in a direction perpendicular to the substrate, forming a highly ordered vertical alignment. Unlike the disordered grain boundary distribution found in traditional polycrystalline films, these vertically aligned grains restrict ion migration to unified paths parallel to the electric field lines. This structural ordering reduces stochastic migration, and the restricted paths ensure high reliability during the conductance modulation process. Furthermore, the uniform composition and smooth surface of the 2D perovskite film promote uniform ion migration within these vertically oriented channels. This facilitates uniform conductance state modulation, thereby ensuring highly linear and symmetric updates of synaptic weights during both the potentiation and depression stages.
